# Survival and ocular preservation in a long-term cohort of Japanese patients with retinoblastoma

**DOI:** 10.1186/s12887-020-1923-7

**Published:** 2020-01-28

**Authors:** Tamaki Ueda, Yuhki Koga, Hiroshi Yoshikawa, Mika Tanabe, Kanako Yamana, Utako Oba, Kentaro Nakashima, Hiroaki Ono, Takuya Ichimura, Shunji Hasegawa, Wakako Kato, Tetsuko Kobayashi, Hideki Nakayama, Yasunari Sakai, Tadamasa Yoshitake, Saiji Ohga, Yoshinao Oda, Shigenobu Suzuki, Koh-Hei Sonoda, Shouichi Ohga

**Affiliations:** 1 Department of Pediatrics, Graduate School of Medical Sciences, Kyushu University, 3-1-1 Maidashi, Higashi-ku, Fukuoka, 812-8582 Japan; 2 Department of Ophthalmology, Graduate School of Medical Sciences, Kyushu University, 3-1-1 Maidashi, Higashi-ku, Fukuoka, 812-8582 Japan; 3 Department of Pediatrics, Yamaguchi University School of Medicine, 1-1-1 Minamikogushi, Ube, Yamaguchi, 755-8505 Japan; 4 Division of Pediatrics, Kyushu Cancer Center, 3-1-1 Maidashi, Higashi-ku, Fukuoka, 812-8582 Japan; 5 Department of Clinical Radiology, Graduate School of Medical Sciences, Kyushu University. 3-1-1 Maidashi, Higashi-ku, Fukuoka, 812-8582 Japan; 6 Department of Pathology, Graduate School of Medical Sciences, Kyushu University, 3-1-1 Maidashi, Higashi-ku, Fukuoka, 812-8582 Japan; 7 Department of Ophthalmic Oncology, National Cancer Center Hospital, 5-1-1 Tsukiji, Chuo-ku, Tokyo, 104-0045 Japan

**Keywords:** *RB1* gene, Radiotherapy, Chemotherapy, Laser-therapy, Cancer predisposition, Blindness

## Abstract

**Background:**

Retinoblastoma is an ocular tumor in infants with cancer predisposition. Treatment of the rare tumor needs to be optimized for ocular preserved survival without second primary malignancy (SPM).

**Methods:**

We studied the outcomes of all patients with retinoblastoma at a tertiary center in 1984–2016, when preservation method changed from radiotherapy (1984–2001) to systemic chemotherapy (2002–2016).

**Results:**

One-hundred sixteen infants developed unilateral- (*n* = 77), bilateral- (*n* = 38), or trilateral-onset (*n* = 1) tumor. Ten (8.6%) had a positive family history, despite a few studies on *RB1* gene. Contralateral disease occurred in one unilateral-onset case. One-hundred eight of 155 eyes (70%) were enucleated. Nine binocular survivors were from 5 bilateral- and 4 unilateral-onset cases. Two survivors received bilateral enucleation. Six deaths occurred; brain involvement (including 3 trilateral diseases) in 4 bilateral-onset, systemic invasion in a unilateral-onset, and SPM (osteosarcoma) in a bilateral-onset case(s). Two others survived SPM of osteosarcoma or lymphoma. The 10-year overall survival (OS: 98.5% vs. 91.3%, *p* = 0.068) and binocular survivors (13.2% vs. 5.2%, *p* = 0.154) between bilateral- and unilateral-onsets did not differ statistically. The 10-year OS and cancer (retinoblastoma/SPM)-free survival (CFS) rates of all patients were 94.9 and 88.5%, respectively. The proportion of preserved eyes did not differ between radiotherapy and chemotherapy eras. The CFS rate of bilateral-onset cases in systemic chemotherapy era was higher than that in radiotherapy era (*p* = 0.042). The CFS rates of bilateral-onset patients with neoadjuvant chemotherapy (upfront systemic therapy for preservation) was higher than those without it (*p* = 0.030).

**Conclusions:**

Systemic chemotherapy and local therapy raised OS and binocular survival rates of bilateral-onset patients similarly to those of unilateral-onset patients. All but one death was associated with a probable germline defect of the *RB1* gene. Neoadjuvant stratified chemotherapy may support the long-term binocular life with minimized risk of SPM.

## Background

Retinoblastoma, which is the most common intraocular tumor of childhood, arises from a mutation in *RB1* [1]. It usually affects young infants; the worldwide incidence of one case per 15,000–20,000 live births [2]. Non-surgical control became the mainstay for ocular preservation after 1980, in order to reduce the risk of late complications and second primary malignancy (SPM) after external beam radiotherapy [3]. In the 1990s, neoadjuvant systemic chemotherapy then emerged as an initial therapy for complete survival and better visual outcome [4]. Intra-ophthalmic artery chemotherapy (IAC) has been also introduced for effective globe salvage and reducing the risk of SPM [5–8].

The first goal in the treatment of retinoblastoma is to prevent extraocular invasion and metastasis of the primary tumor. In developing countries, this tumor is still diagnosed at an advanced stage [9]. The majority of patients having metastatic retinoblastoma die within approximately 6 months [10]. The annual incidence of retinoblastoma in Japan is estimated to be < 100 per year, and the treatment regimen has yet been unified [11]. Even in developed countries, very young infants are hard to complete both IAC and neoadjuvant systemic chemotherapy (upfront intravenous chemotherapy) in a single institution [6, 12]. There is little information on the late effects of systemic chemotherapy on mortality and morbidity along with the risk of developing SPM in association with a germline mutation of *RB1*.

To search for better practices for retinoblastoma, we retrospectively studied the final outcomes of patients in our tertiary institution over three decades, focusing on the survival and ocular preservation of survivors. The best treatment goal was discussed with respect to cancer-free and blindness-free survival of patients with retinoblastoma.

## Methods

### Data collection

**Fig. 1:**
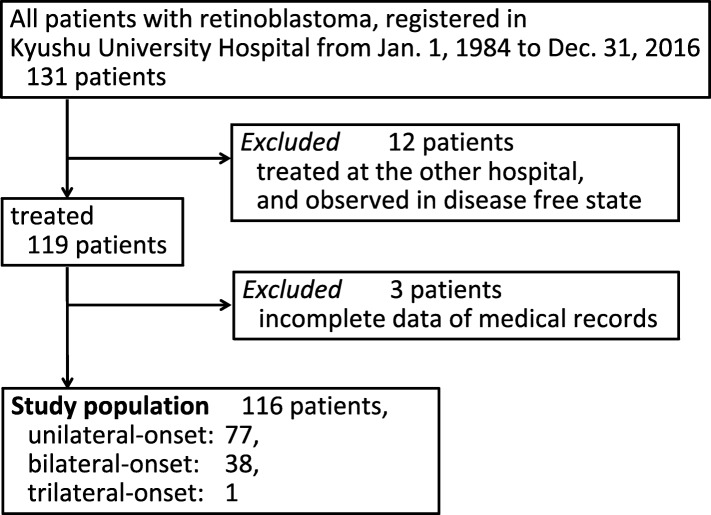
Flowchart of the clinical cohort of patients with retinoblastoma in Kyushu University Hospital, Japan

One-hundred thirty-one patients who received a diagnosis of retinoblastoma were consecutively registered in Kyushu University Hospital from 1984 to 2017 (Fig. 1). The inclusion criteria were patients who received a diagnosis and treatment in Kyushu University, and who underwent IAC in the National Cancer Center Hospital (to standardize the treatment modality). Patients with underlying diseases were excluded from the study. Twelve patients who were only referred to our hospital for follow-up after treatment elsewhere, and 3 patients for whom no information was available were excluded. A total of 116 patients with 155 affected eyes were enrolled in the study. The following data were collected from the medical records and questionnaires distributed to attending doctors and/or families: age at the diagnosis, sex, laterality, treatment (radiotherapy, systemic chemotherapy, and/or local therapy), SPM, family history. The primary endpoint in the study is overall survival (OS), and the secondary ones were cancer (retinoblastoma/SPM)-free survival (CFS) and ocular outcome (number of preserved eyeballs). This study was certified by the Institutional Review Board of Kyushu University (#29–244).

### Diagnosis and classification

The diagnosis of retinoblastoma was clinically and histopathologically made by ophthalmologists and pathologists. The stage of extraocular disease was classified according to the International Retinoblastoma Staging System (IRSS) [13]. Intraocular disease was classified by Reese and Ellsworth (R-E) system or the Intraocular Classification of Retinoblastoma (ICRB) [14]. Staging to determine the treatment choice in each patient was independently assessed by two expert ophthalmologists in different institutions.

### Treatment modality

Intravenous chemotherapy was applied for cases with IRSS stage II-IV extraocular disease, or progressive intraocular disease without vitreous seeding. For local therapy, laser irradiation or cryotherapy or direct vitreous injection of melphalan were performed in Kyushu University Hospital. IAC and brachytherapy were performed by the National Cancer Center Japan. Brachytherapy was included as local therapy because of the report on the negligible effect on the development of SPM [15].

**Fig. 2:**
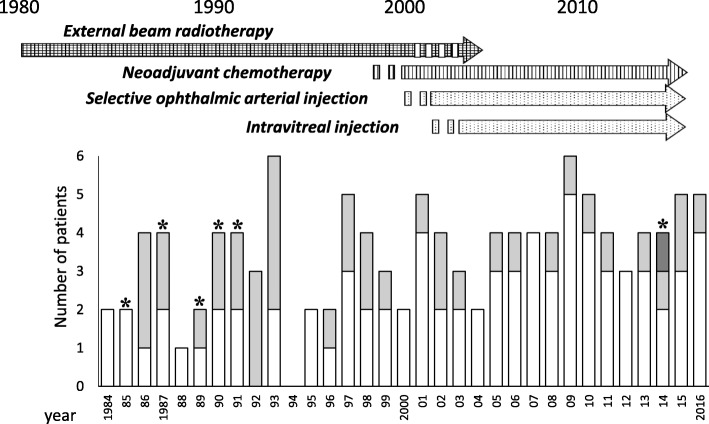
The number of patients with retinoblastoma and the changing modalities of the first-line therapy for ocular preservation. White, gray, and dark gray boxes represent unilateral-, bilateral-, and trilateral-onset patients, respectively. Asterisks indicate deceased cases (*N* = 1 for each)

External beam radiotherapy was performed for the preservation of one eye in bilateral cases or for the control of extraocular disease until 2002 (Fig. 2). Intravenous cancer chemotherapy was used for the control of extraocular lesions until 2000, using cyclophosphamide (CPM), vincristine (VCR), pirarubicin (THP-ADR), and cisplatin (CDDP). The combination chemotherapy of VCR, etoposide, and carboplatin (VEC therapy) as local therapy was introduced as adjuvant and neoadjuvant therapy in 1998 and 2002, respectively. The standard dosages of 3 drugs were used; VCR (1.5 mg/m^2^, day 1), etoposide (150 mg/m^2^, day 1, 2), carboplatin (560 mg/m^2^, day 1). The administered dose was weight-converted to 1 m^2^ = 30 kg for patients weighing < 10 kg. The dose was reduced by 50–75% of reduced for newborn infants or patients with a poor condition. The number of cycles of VEC was individualized according to the aforementioned classifications, pathological consequences, appearance of the fundus, extension, and chemoresponse of the tumor.

The first-line therapy for patients with unilateral disease was enucleation prior to 1987, local therapy for ocular preservation since 1988, and neoadjuvant therapy from 2002, according to the stage. The first-line therapy for bilateral cases was enucleation of the advanced stage eye and preservation of the eye on the other side prior to 1997, and neoadjuvant therapy with local therapy from 1998. Multidrug chemotherapy was administered as first-line therapy for central nervous system (CNS) involvement or distant metastasis throughout the study period.

### Statistical analyses

The chi-squared test or Fisher’s exact test was used to compare categorical variables. The Mann-Whitney U test was used to compare the mean values of continuous variables. The OS and CFS rates with 95% confidence intervals (CI) were estimated by the Kaplan–Meier method, and assessed by a log-rank test. OS was defined as the period from the date of the diagnosis of retinoblastoma until death of any cause or the final observation. CFS was defined as the period from the date of the diagnosis of retinoblastoma until the date of the “*progression*” of retinoblastoma, the diagnosis of SPM, for death from any cause or the final observation. The term “*progression*” was defined as growth, regrowth, vitreous dissemination in the affected eyeball, or extraocular invasion or metastasis of the primary tumor. Transient minimal growth was excluded from “*progression*”. Contralateral ocular development and CNS involvement during the study period were considered “*progression*”, because most patients did not undergo a mutation study of the germline *RB1* gene and CNS diseases were diagnosed by imaging but not all histopathological studies. The observation period was calculated from the diagnosis to the last contact (the date at visit or questionnaire response) with patients and/or their families directly. All results were updated to September 30, 2017. A logistic regression model was used to investigate factors associated with death or the occurrence of SPM. *P*-values of < 0.05 were considered to indicate statistical significance. Statistical analyses were performed with Excel statistical software and JMP software (ver.13 SAS institute, Cary, NC, U.S.A) programs.

## Results

### Demographics and the first-line treatment

They consisted of 77 unilateral- (66%), 38 bilateral- (33%), and one trilateral-onset patients (Table 1). Bilateral-onset patients had a higher rate of family history of retinoblastoma, a younger age at diagnosis and a longer observation period than the unilateral-onset ones. Figure 2 shows the historical changes in the patient number and the initial non-surgical treatment modality. The annual incidence was average 4 cases per year. The ratio of bilateral- to unilateral-onset patients in radiotherapy era (1984–2001) was higher than that in preservation chemotherapy era (2002–2016) (44% vs. 22%, *p* = 0.029). The mortality rates decreased from 9.1% in the former to 1.6% in this century. The trilateral-onset was only one death after 2000. The proportion of preserved eyes did not increase from the former to this century (33 to 26%). Contralateral disease occurred only in one unilateral-onset patient. (Additional file 1: Figure S1A).

**Table 1:** Demographics and initial treatment in 116 patients at diagnosis of retinoblastoma

	All patients	Unilateral	Bilateral	Trilateral	*p*-value ^a)^
Number of patients	116	77	38	1	
eyes	155	77	76	2	
Male / female	58 / 58	36 / 41	21 / 17	1 / 0	0.432
Positive family history ^b)^	10	2	8	0	0.002
Age at diagnosis ^c)^, month	13 (0–101)	20 (0–101)	5 (0–28)	18	3.17 × 10-
Observation period ^b)^, month	133 (10–403)	113 (10–403)	188 (15–370)	18	0.026
Initial treatment					
Enucleation alone	69	69	0	0	
Local therapy alone	6	0	6	0	
External beam radiotherapy	19	2	17	0	
Systemic chemotherapy	22	6	15	1	

^a)^ Statistical comparisons between patients with unilateral tumors and those with bilateral tumors were

performed, using chi-square test, Fisher’s exact test and Mann-Whitney U test

^b)^ The germline mutation of *RB1* was studied in only a few patients

^c)^ Each value represents the median age or observation period (months), with the range in parenthesis

### Treatment course and outcomes

The treatments, deaths and ocular preservation in survivors are shown in Table 2. During the observation (median 133 months; range, 10–403), 6 deaths occurred from CNS (including 3 trilateral) disease in 4 bilateral-onset, metastasis in a unilateral-onset, and SPM (osteosarcoma) in a bilateral-onset case(s). Two survivors became blind for both enucleations. The binocular survivor’s rate of bilateral-onset (13.2%) was high compare with unilateral-onset ones (5.2%, *p* = 0.154). Ten (6 bilateral- and 4 unilateral-onset) received IAC and systemic chemotherapy (Table 2, Additional file 1: Figure S1A, and Figure S1B).

**Table 2:** All treatments, deaths, and ocular preservations in survivors

Disease	Patients	Treatments	Number of Patients	Death	Binocular Survivors (%)	
Trilateral-onset *n* = 1	*n* = 1	RT and Systemic chemotherapy	1	1 (Pt 2)	0	(0%)
Bilateral-onset *n* = 38	*n* = 38	Enucleation and/or Local therapy alone	5	0	1 / 5	(25%)
		Enucleation and/or Local therapy with RT	8	2 (Pt 3, Pt 6)	2 / 6	(33%)
		Systemic chemotherapy	25	2 (Pt 5, Pt 1)	0 / 8^a^	(0%)
		with RT (and IAC)	10 (1)	2	2/ 15	(13%)
		w/o RT (and IAC)	15 (5)	0	0	(0%)
				4/38 (10.5%)	5/38	(13.2%)
Unilateral-onset *n* = 77	*n* = 77	Enucleation and/or Local therapy alone	52	0	0 / 52	(0%)
		Enucleation and/or Local therapy with RT	2	0	0 / 2	(0%)
		Systemic chemotherapy	23	1 (Pt 4)	0 / 4	(0%)
		with RT	5	1	4 / 18	(22%)
		w/o RT (and IAC)	18 (4)	0	0 / 52	(0%)
				1/77 (1.3%)	4/77	(5.2%)

^a^Two survivors were blind due to binocular loss

Detailed treatments of unilateral- (**A**) and bilateral-onset patients (**B**) are shown in Additional file 1: Figure S1. Final outcomes are represented by death (black box, *n* = 5), binocular (double bold frame, *n* = 2) or monocular enucleation (single bold frame, *n* = 103). According to the reported risk of SPM [16], patients with systemic chemotherapy (*n* = 33) and/or external beam radiotherapy (*n* = 25) may have an increased risk of cancer predisposition (gray box). All survivors achieved complete remission (CR) at the time of last observation.

In 77 unilateral-onset cases (Additional file 1: Figure S1A), 69 underwent enucleation and 8 received preservation therapy. After enucleation, the presence of residual tumor cells was microscopically suspected in 17 cases. One developed a contralateral lesion 5 months later; the other died of dissemination 27 months after diagnosis (Pt-4, Table 3). Fifty of 69 enucleated patients attained a CR without additional therapy, one of whom developed T-cell lymphoma 82 months after the diagnosis of retinoblastoma (Pt-8, Table 4). Among 17 cases with microscopic invasion suspected in the enucleated site, 15 obtained a CR after external beam radiotherapy and/or systemic chemotherapy and 2 attained a CR after no additional therapy. Among 8 cases with local therapy, 6 received neoadjuvant chemotherapy and two resulted in enucleation. Two others underwent enucleation after external beam radiotherapy.

**Table 3:** Characteristics, treatment and secondary neoplasms of deceased patients

Pt	Sex	Age at diagnosis, death		Observation period	Onset	Invasion^a^	Treatment for retinoblastoma RT /Initial sys-chemo/Enucl				Family history	Secondary malignancy	Causes of death	Onset
1	male	21 m,	37 m	15 m	bilateral	CNS (tri.)	yes^b^	no		Lt	NR	no	trilateral disease	1987
2	male	17 m,	37 m	18 m	trilateral	CNS (tri.)	yes ^b^	yes		no	no	no	trilateral disease	2014
3	male	0 m,	24 m	23 m	bilateral	CNS (tri.)	yes	no		Rt	NR	no	trilateral disease	1989
4	male	21 m,	49 m	27 m	unilateral	systemic	yes ^b^	no		Lt	no	no	systemic disease	1985
5	female	1 m,	93 m	92 m	bilateral	CNS (meta.)	yes	no	Pr →	Rt	yes	no	CNS metastasis	1990
6	male	7 m,	298 m	290 m	bilateral	CR → 2nd	yes	no		Lt	no	Os	secondary Os	1991

^a^ Invasion means the sites affected during the advanced disease course

^b^ External beam radiotherapy for metastasis

*m* months; *meta* metastasis; *tri* trilateral; *Lt* left; *Rt* right; *Pr* preservation; *NR* not recorded; *RB* retinoblastoma; *CR* complete remission; *Os* osteosarcoma; *RT* external beam radiotherapy

**Table 4:** Characteristics of patients with secondary neoplasm

Pt	Sex	Age at diagnosis		Duration months	Disease course and treatment		Family history	Outcome, observation period	
		RB, second malig.			Retinoblastoma	second malignancy			
6	male	7 m	19	223	bilateral→Enuc. + R,L → CR	Os→C + S + R → PD	no	died of Os,	290 m
7	male	22 m	7 y	74	bilateral→prEnuc. + R,C,L → CR	Os→C + S → CR	no	alive on CR,	235 m
8	male	19 m	8 y	82	unilateral→Enuc. → CR	Lym → C → CR	no	alive on CR,	167 m

*Enuc* enucleation; *prEnuc* ocular preservation but subsequent enucleation; *CR* complete remission; *L* local therapy; *C* systemic chemotherapy; *S* surgical intervention; *R* external beam radiotherapy; *Os* osteosarcoma; *Lym* T-cell lymphoma

In 38 bilateral-onset cases (Additional file 1: Figure S1B), 27 first underwent one enucleation, and 11 received local therapy for binocular preservation. Three of 27 patients died. Among 11 cases with preservation therapy, only one died and all survivors escaped blindness. Among 27 enucleated cases, 3 were observed alone, 9 received systemic chemotherapy and 15 did external beam radiotherapy. All 12 non-irradiated cases attained a CR. On the other hand, 3 of 15 cases that received radiotherapy died of progression (Pt-1 and Pt-3, Table 3) or osteosarcoma (Pt-6, Table 3**,** Table 4). External beam radiotherapy was given to the ocular and metastatic disease. The remaining 12 cases obtained a CR, but one developed osteosarcoma (Pt-7, Table 4) and 2 required binocular enucleation. Among 11 cases that received local therapy for binocular preservation, 6 resulted in enucleation and one died of progression (Pt-5, Table 3). Only a case attained binocular preservation without systemic chemotherapy or radiotherapy.

### Deceased cases and second malignancy

Six deceased and 3 SPM cases are summarized in Table 3 and Table 4, respectively. Two bilateral-onset cases had trilateral disease 5 and 23 months after the first diagnosis, respectively (Pts-1, 3). All 3 cases with trilateral lesions died within 2 years (Pts-1~3). Osteosarcoma developed in 2 bilateral-onset cases (Pts-6 and 7). T-cell lymphoma did in a unilateral-onset case without no radiotherapy or systemic chemotherapy. Five advanced cases with CNS or bone marrow involvement died within 100 months after the diagnosis (Additional file 1: Figure S2).

### Survival, risk of developing cancer, and ocular outcomes

**Fig. 3:**
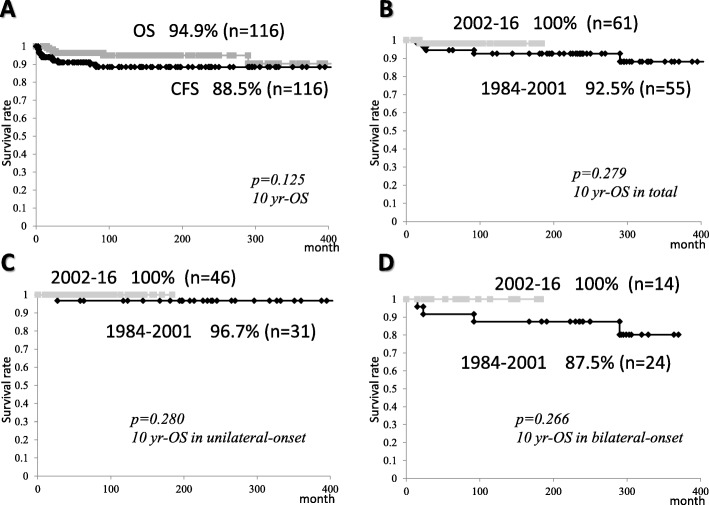
The cumulative probability of survival rates of patients after the diagnosis. Each value was assessed by a log-rank test. Kaplan-Meier curves showing **a** the 10-year overall survival (OS) versus cancer (retinoblastoma/second primary malignancy [SPM])-free survival (CFS) rates of all patients, **b** the 10-year CFS rates of all patients in 1984–2001 versus 2002–2016, **c** the 10-year CFS rates of bilateral-onset patients in 1984–2001 versus 2002–2016, **d** the 10-year CFS rates of unilateral-onset versus bilateral-onset patients

**Fig. 4:**
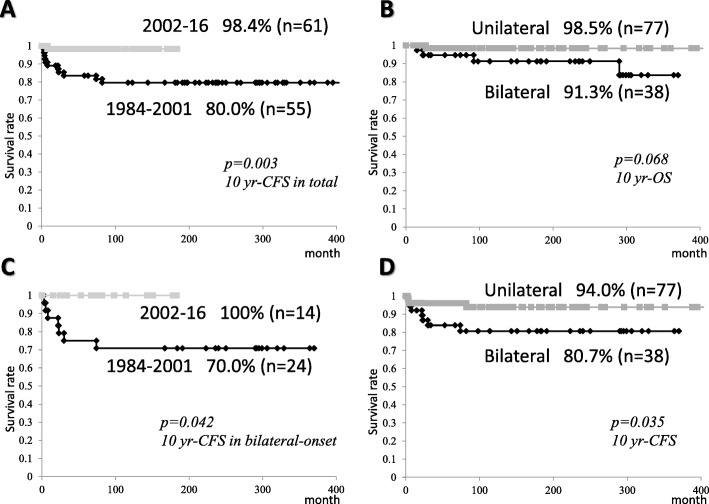
The cumulative probability of OS rates of patients after the diagnosis. Each value was assessed by a log-rank test. Kaplan-Meier curves showing **a** the 10-year OS rates of all patients in 1984–2001 versus 2002–2016, **b** the 10-year OS rates of unilateral-onset patients in 1984–2001 versus 2002–2016, **c** the 10-year OS rates of bilateral-onset patients in 1984–2001 versus 2002–2016, and **d** the 10-year OS rates of unilateral-onset versus bilateral-onset patients

The 10-year OS rates (median ± SE%) and CFS rates of all patients were 94.9 ± 2.3% and 88.5 ± 3.2%, respectively (Fig. 3a). The 10-year CFS rates of all and bilateral-onset cases, but not unilateral-onset cases in 2002–2016 were higher than those in 1984–2001 (98.4 ± 1.6% vs. 80.0 ± 5.5%, *p* = 0.003, 100 ± 0% vs. 70.0 ± 9.3%, *p* = 0.042, respectively) (Fig. 4a, c). On the other hand, the 10-year OS rates of all, unilateral-onset, and bilateral-onset cases did not differ between the era of radiotherapy (1984–2001) and chemotherapy (2002–2016) (Figs. 3a, 4b, 3d). The 10-year CFS rates of bilateral-onset cases was lower than seen in unilateral ones (94.0 ± 3.1% vs. 80.7 ± 6.6%, *p* = 0.035) (Fig. 4d), although the difference of the 10-year OS rates did not reach the statistical significance (98.5 ± 1.5% vs. 91.3 ± 4.9%, *p* = 0.068) (Fig. 4b).

**Fig. 5:**
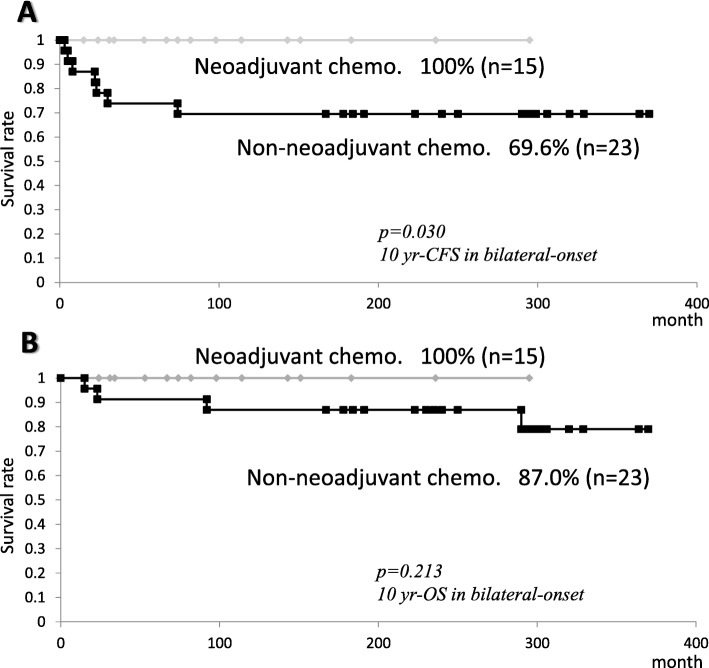
Kaplan-Meier curves showing (**a**) the 10-year DFS rates and (**b**) the 10-year OS rates of bilateral-onset patients who received neoadjuvant chemotherapy versus no neoadjuvant chemotherapy

The 10-year CFS, but not OS, rates of cases with neoadjuvant chemotherapy were higher than those without it (100% vs. 69.6%, *p* = 0.003) (Fig. 5a, b), while the final proportion of binocular survivors did not differ between two groups (13.3% vs. 13.0%, *p* > 0.999). The Cox-hazards model indicated that neoadjuvant chemotherapy (OR 44.4, 95%CI 3.65–540, *p* = 0.003) and a positive family history (OR 13.1, 95%CI 1.06–162, *p* = 0.045) were independent variables that discriminated patients who attained radiotherapy-free, disease-free and SPM-free survival after binocular salvage (Table 5).

**Table 5:** Multivariable analysis for the association variables discriminating non-irradiated survivors with disease-free both eyes

Variables	Odds ratio	95% confidence interval lower-upper limit	*P*-value
Neoadjuvant chemotherapy	44.4	3.65–540	0.0029
Positive family history^a^	13.1	1.06–162	0.045
Unilateral RB at diagnosis	7.32	0.68–84.2	0.11
male	2.63	0.35–19.9	0.35

Survivors in whom both eyes were disease-free (*n* = 7) vs. others (*n* = 83) who received enucleation

or external beam radiotherapy (*n* = 26)

^a^Either parents or sibling(s) received a diagnosis of retinoblastoma

## Discussion

This is the first and largest cohort of retinoblastoma patients treated with systemic chemotherapy in Japan. The CFS rates of binocular-onset patients improved in systemic chemotherapy era. Upfront systemic chemotherapy with local therapy has sustained the high OS and CSF rates but limitedly ocular preservation. Despite the high CFS rates over 85%, 5 of 6 deaths were associated with a probable germline defect of *RB1* gene because of their trilateral diseases and SPM. These results stressed the need of stratified protocol of upfront chemotherapy according to cancer-predisposing grade of patients.

In the United States, 75% of children with retinoblastoma have advanced or refractory disease that cannot be cured with focal treatments alone, and thus require external beam radiotherapy, systemic chemotherapy, or enucleation [17]. In developing countries [18], systemic chemotherapy may have a greater utility in practice for not only preservation but also saving lives because of the delayed diagnosis and lower availability of focal treatments, including IAC. In Japan, systemic preservation chemotherapy has been conducted by pediatric oncologists in collaboration with ophthalmologists. In the present study, the proportion of unilateral cases in the non-preservation era (enucleation and radiotherapy, 1984–2001) was lower than seen in the preservation era (systemic chemotherapy, 2002–2016). This may be explained by the early diagnosis and intervention in Japan of this century. It also represents the centralization of advanced cases or preservable cases to our hospital after 2000, when it was officially designated as the largest center for pediatric cancer treatment in western Japan. The present cohort was completed with long-term collaboration between pediatricians and ophthalmologists in the Tokyo (IAC) and Kyushu (systemic chemotherapy) centers, with the primary endpoint of saving life, with globe salvage and the preservation of vision in the affected eyes. Although it previously allowed most eyes to be salvaged from enucleation, IAC alone does not replace systemic chemotherapy for the control of refractory disease [5]. This is the first report demonstrating the effects of upfront systemic chemotherapy on cancer-free survival, it also reports—albeit insufficiently—the binocular outcomes of Japanese survivors with retinoblastoma.

The major concern is the effect of systemic chemotherapy on the tumor growth and secondary tumorigenesis. Extraocular metastasis occurs even if careful local therapy is given to the refractory case individually. Adjuvant systemic chemotherapy prevents the development of extraocular metastasis [19]. In the present study, no cases receiving neoadjuvant chemotherapy (with an exception of trilateral disease) died of progressive disease or SPM; however, the refractory side-enucleation was inevitable for bilateral-onset cases. In cases of neoadjuvant chemotherapy, no trilateral disease occurred during the observation period. Chemoreduction could prevent or delay the onset of trilateral retinoblastoma, although the prophylactic effects have not been verified [20].

During our long observation, 3 of 116 patients (2.6%) suffered from SPM over 5 years after the diagnosis. In a Japanese cohort of 754 patients treated from 1964 to 2007 [21], 21 patients (2.8%) developed SPM. Thereafter, the cumulative incidence rate of SPM increased to 4.3% at 20 years and 19.1% at 40 years. On the other hand, a pediatric oncology study group in Japan [22] reported that the cumulative incidence of SPM at 20 years was highest in patients with osteosarcoma (13.1%), followed by those with hepatoblastoma (8.4%) and retinoblastoma (6.6%). In this setting, the current neoadjuvant therapy needs to be optimized in terms of the balance between the intensity for preservation and the much longer-term cancer risk over 20 years.

Treatments for metastatic or trilateral disease are problematic for survival [23]. The other issue is the intensity of neoadjuvant chemotherapy for newborns and very young infants. In our cohort, 16 patients received the diagnosis within 3 months after birth. We conducted reduced-dose VEC chemotherapy for them. To reduce the late effects, single-agent systemic chemotherapy may be recommended for young infant as “bridge therapy” to provide time for the infant to grow to a size that permits successful arterial cannulation, at which time IAC can be performed [24]. On the other hand, standard chemotherapy protocols occasionally failed to treat the neonatal retinoblastoma because of the lack of a significant vascular supply in smaller tumor foci [25].

Recently, molecular targeted drugs and gene therapy have been reported as the promising therapeutic option for advanced cases and/or young infants with retinoblastoma [26–28]. Rare deaths in our institution might account for the probable germline defect of *RB1* gene, although most patients received no genetic study. To pursue a better quality of life, in addition to a stratified neoadjuvant chemotherapy protocol, neonatal screening is being more actively promoted for high-risk families in Japan under collaboration between ophthalmologists and pediatricians [29].

The present study has some limitations. First, the analysis based on international classification was not possible due to insufficient information regarding tumor stage. Second, the genetic study was not done in most cases because of personal information and health insurance system in Japan. Third, the follow-up time might be insufficient to determine the risk of SPM in patients with retinoblastoma.

## Conclusion

The survival and ocular outcomes of bilateral cases improved in the era of systemic chemotherapy for preservation. The neoadjuvant chemotherapy may support their long-term outcomes with less impact on the risk of developing SPM.

## Supplementary information

**Additional file 1: Figure S1.** (Online Resource) The treatment courses and outcomes of unilateral-onset retinoblastoma (A) and bilateral-onset retinoblastoma (B). White box indicates observation and/or local therapy. Gray box indicates *“the second primary malignancy risk-inducible therapy”* of external beam radiotherapy or systemic chemotherapy. Black box indicates death. Single and double bold frames indicate monocular and binocular enucleation, respectively. Star symbol indicates second primary malignancy (SPM). CR: complete remission, Chemo Tx: systemic chemotherapy, BM: bone morrow, RT: external beam radiotherapy, PD: progressive disease, IAC: Intra-ophthalmic artery chemotherapy. Each patient number corresponds to the deceased case in **Figure S2.** **Figure S2.** (Online Resource) The detailed treatment course from diagnosis to death of all 6 deceased patients. RT(m): external beam radiotherapy for metastasis, RT(e): external beam radiotherapy for eye, RT(s): external beam radiotherapy for SPM, Chemo: systemic chemotherapy, Local: local therapy for preservation, CSF: cerebrospinal fluid, BM: bone marrow, CNS: central nervous system.

## Data Availability

Complete raw data is available on request. Kindly contact the corresponding authors for raw data.

## Abbreviations

CDDP: Cisplatin
CFS: Cancer- free survival
CNS: Central nervous system
CPM: Cyclophosphamide
IAC: Intra-arterial chemotherapy
OS: Overall survival
RT: External beam radiotherapy
SPM: Second primary malignancy
THP-ADR: Pirarubicin
VCR: Vincristine
VEC: Vincristine-etoposide-carboplatin
