# Supplementary material for: Survival and ocular preservation in a long-term cohort of Japanese patients with retinoblastoma
Source: BMC Pediatr. 2020 Jan 28;20:37. doi: 10.1186/s12887-020-1923-7 (PMC6986142; doi:10.1186/s12887-020-1923-7)
Supplement: Supplementary file 1 — Additional file 1: Figure S1. (Online Resource) The treatment courses and outcomes of unilateral-onset retinoblastoma (A) and bilateral-onset retinoblastoma (B). White box indicates observation and/or local therapy. Gray box indicates “the second primary malignancy risk-inducible therapy” of external beam radiotherapy or systemic chemotherapy. Black box indicates death. Single and double bold frames indicate monocular and binocular enucleation, respectively. Star symbol indicates second primary malignancy (SPM). CR: complete remission, Chemo Tx: systemic chemotherapy, BM: bone morrow, RT: external beam radiotherapy, PD: progressive disease, IAC: Intra-ophthalmic artery chemotherapy. Each patient number corresponds to the deceased case in Figure S2. Figure S2. (Online Resource) The detailed treatment course from diagnosis to death of all 6 deceased patients. RT(m): external beam radiotherapy for metastasis, RT(e): external beam radiotherapy for eye, RT(s): external beam radiotherapy for SPM, Chemo: systemic chemotherapy, Local: local therapy for preservation, CSF: cerebrospinal fluid, BM: bone marrow, CNS: central nervous system. [file 12887_2020_1923_MOESM1_ESM.ppt]

## Slide 1
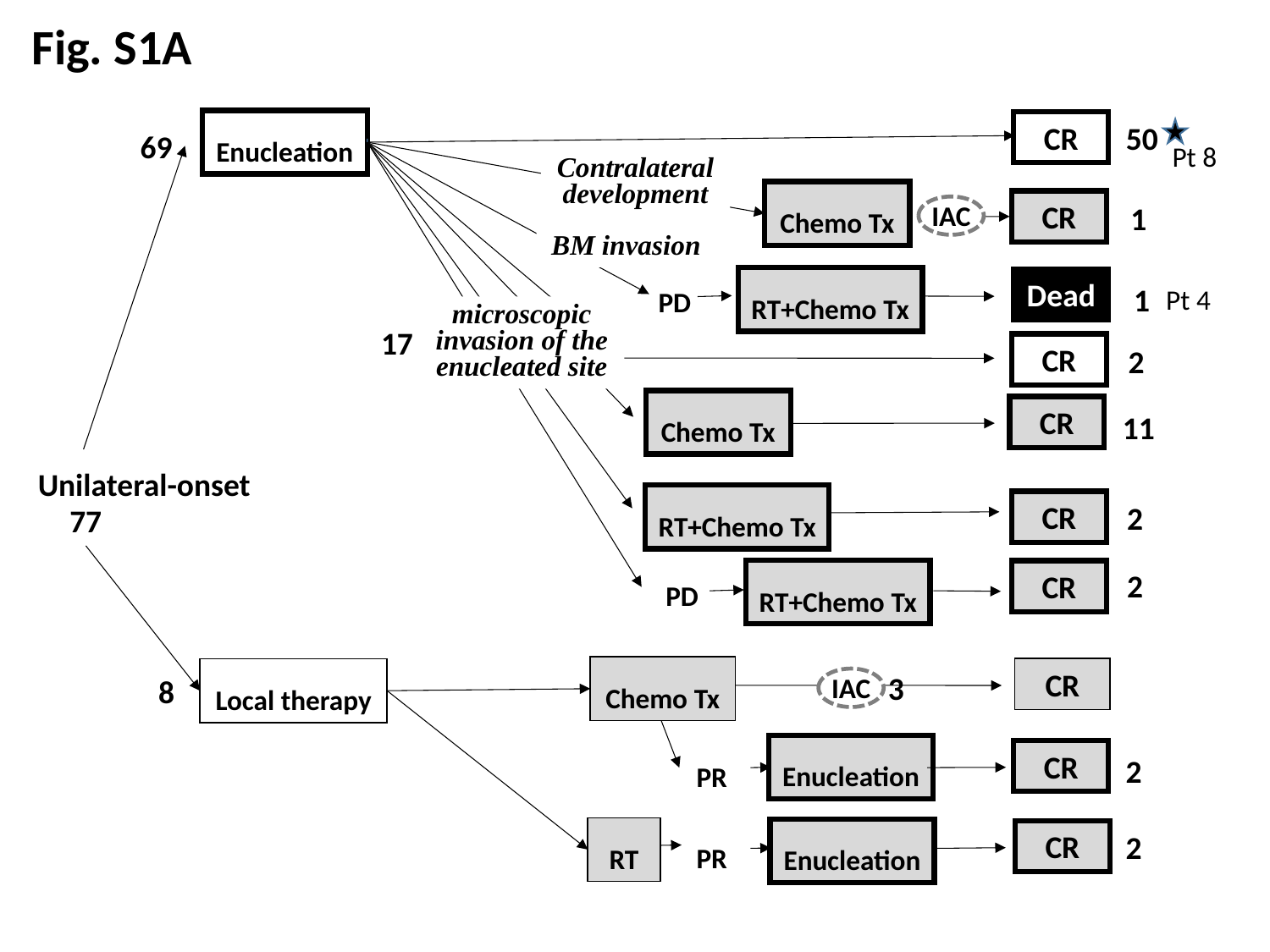

Fig. S1A
Enucleation
CR
50
69
Pt 8
Contralateral development
Chemo Tx
CR
IAC
1
BM invasion
PD
RT+Chemo Tx
Dead
1
Pt 4
microscopic
invasion of the enucleated site
17
CR
2
Chemo Tx
CR
11
Unilateral-onset
RT+Chemo Tx
CR
2
77
PD
2
RT+Chemo Tx
CR
Chemo Tx
CR
Local therapy
3
IAC
8
Enucleation
PR
CR
2
PR
RT
Enucleation
CR
2

## Slide 2
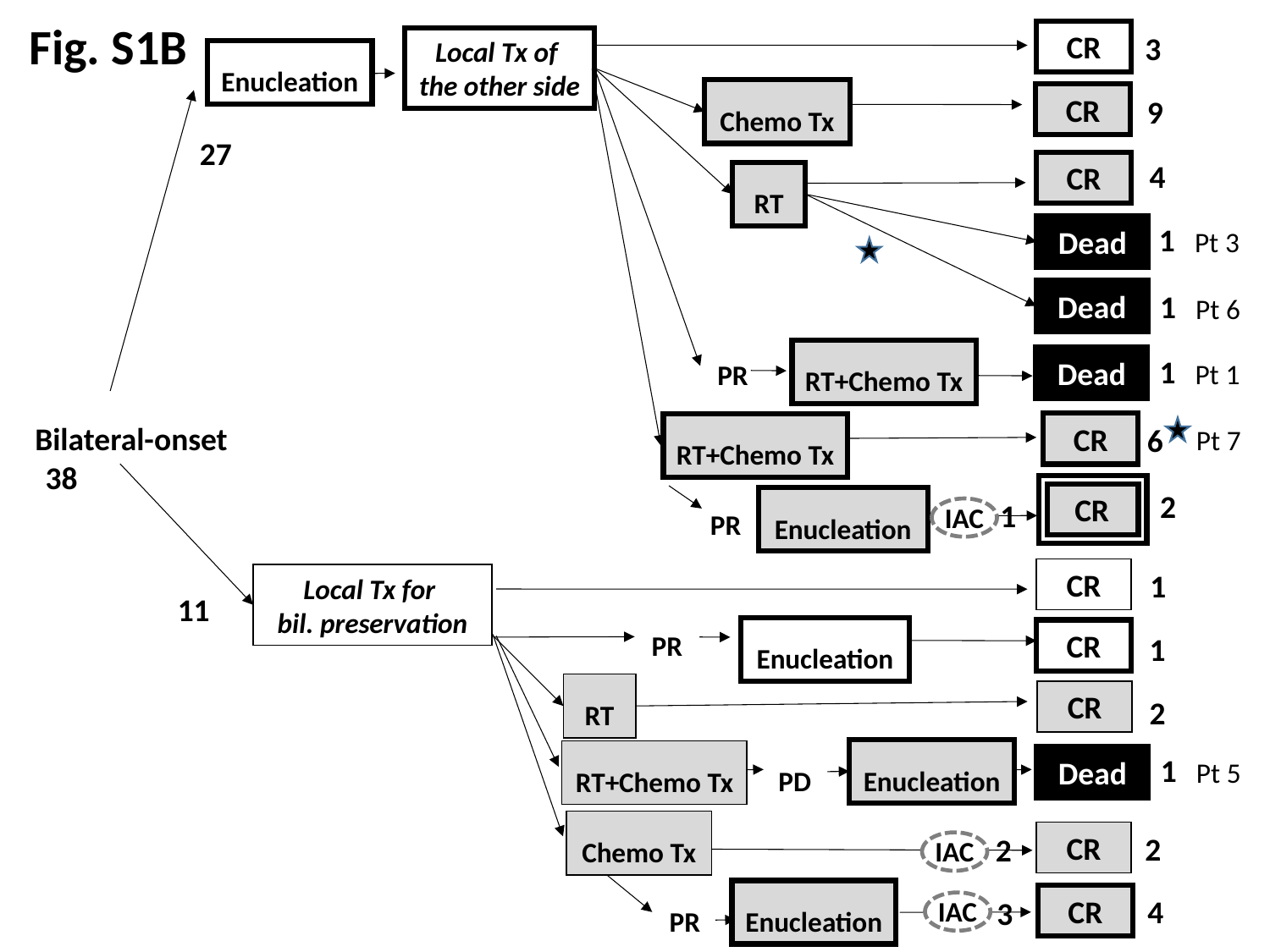

Fig. S1B
CR
3
Local Tx of
the other side
Enucleation
Chemo Tx
CR
9
27
4
CR
RT
1
Dead
Pt 3
1
Dead
Pt 6
RT+Chemo Tx
PR
1
Dead
Pt 1
RT+Chemo Tx
Bilateral-onset
CR
6
Pt 7
38
2
CR
PR
Enucleation
1
IAC
CR
1
Local Tx for
bil. preservation
11
PR
Enucleation
CR
1
RT
CR
2
Enucleation
PD
RT+Chemo Tx
1
Dead
Pt 5
Chemo Tx
CR
2
2
IAC
PR
Enucleation
CR
4
3
IAC

## Slide 3
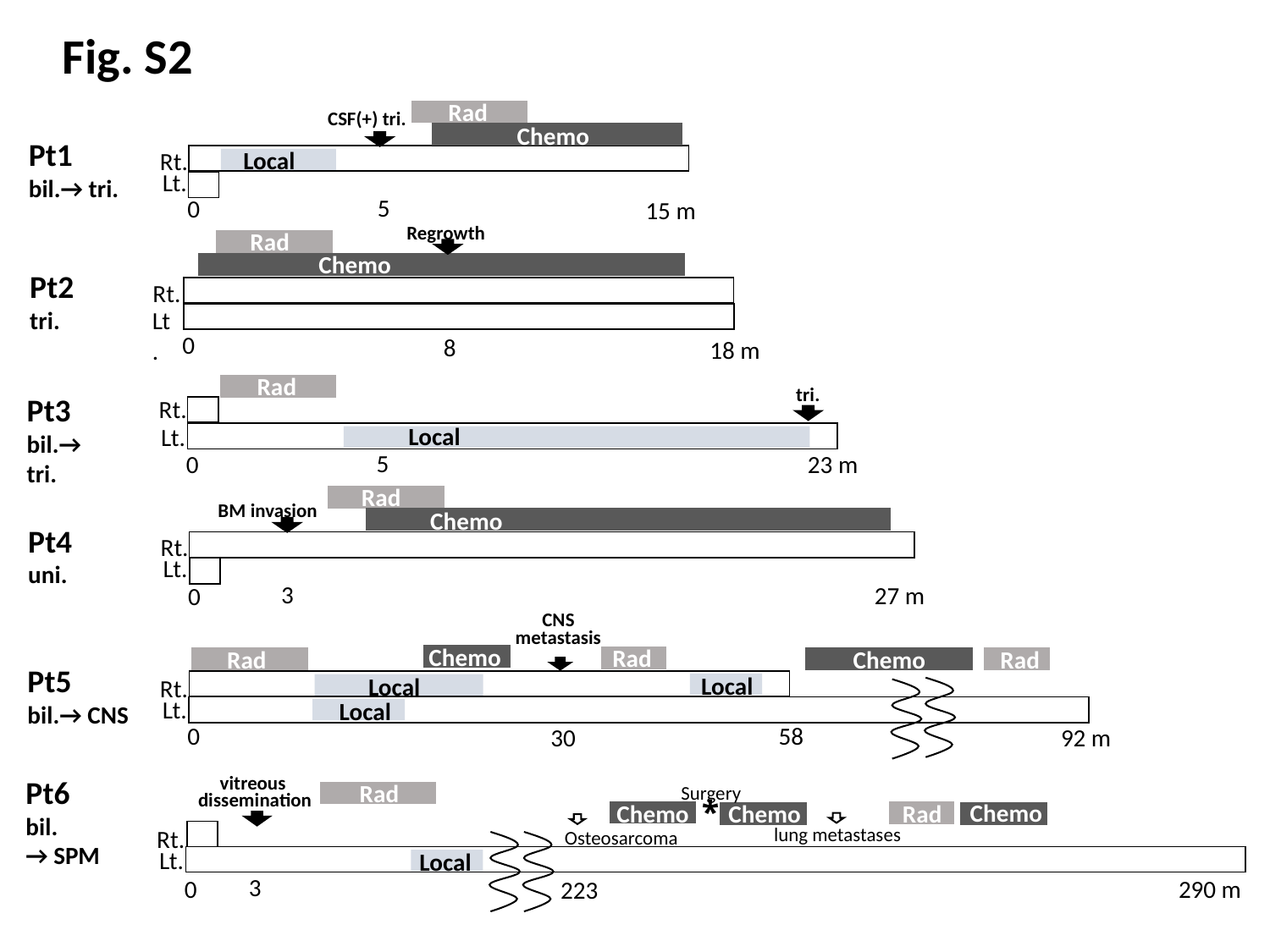

Fig. S2
Rad
CSF(+) tri.
Chemo
Pt1
bil.→ tri.
Local
Rt.
Lt.
5
0
15 m
Regrowth
Rad
Chemo
Pt2
tri.
Rt.
Lt.
0
8
18 m
Rad
tri.
Pt3
bil.→ tri.
Rt.
Local
Lt.
5
0
23 m
Rad
BM invasion
Chemo
Pt4
uni.
Rt.
Lt.
3
27 m
0
CNS metastasis
Chemo
Rad
Rad
Chemo
Rad
Pt5
bil.→ CNS
Local
Local
Rt.
Lt.
Local
0
58
92 m
30
Pt6
bil.
→ SPM
vitreous
dissemination
Rad
Surgery
*
Chemo
Chemo
Chemo
Rad
Rt.
Osteosarcoma
lung metastases
Lt.
Local
3
0
290 m
223
